# Association between the XRCC1 polymorphisms and clinical outcomes of advanced NSCLC treated with platinum-based chemotherapy: a meta-analysis based on the PRISMA statement

**DOI:** 10.1186/s12885-017-3487-y

**Published:** 2017-07-25

**Authors:** Dan-Juan Li, Dong Xiao

**Affiliations:** 1 Department of Oncology, Nanfang Hospital, Southern Medical University, Guangzhou, 510515 China; 2 Cancer Research Institute, Southern Medical University, Guangzhou, 510515 China

**Keywords:** XRCC1, Polymorphism, Lung cancer, Platinum, Meta-analysis

## Abstract

**Background:**

Base excision repair (BER) pathway is a DNA repair pathway that is important in carcinogenesis and in response to DNA-damaging chemotherapy. XRCC1 is one of important molecular markers for BER. So far, the role of XRCC1 polymorphisms with clinical outcomes of advanced NSCLC treated with platinum-based chemotherapy is inconclusive. To explore the relationship between XRCC1 polymorphisms and platinum-based chemotherapy in advanced NSCLC patients, we performed this meta-analysis.

**Methods:**

Crude odds ratios (ORs), Cox proportional hazard ratios (HRs) with the corresponding 95% confidence intervals (CIs) were adopted to assess the strength of association between XRCC1 polymorphisms and response rate, Overall survival (OS) and progression free survival (PFS) of advanced NSCLC treated with platinum-based chemotherapy. Q test and *I* ^2^ test were used for the assessment of heterogeneity. Subgroup analyses were conducted when heterogeneity exists. Begg’s funnel plots and Egger’s linear regression test were used to estimate publication bias. Sensitivity analysis was performed to evaluate the stability of the result.

**Results:**

A total of 19 studies including 2815 individuals were eligible for the analysis, results showed XRCC1 194Arg allele was negatively associated with the objective response rate relative to 194Trp, and results of homozygous model, dominant model and heterozygous model suggested a gene dosage effect negative correlation between 194Arg allele and objective response rate(ArgArg vs TrpTrp: OR = 0.64(95%CI: 0.44-0.91); ArgArg + TrpArg vs TrpTrp: OR = 0.79(95%CI: 0.57-1.11); TrpArg vs TrpTrp: OR = 1.05(95%CI: 0.73-1.51)). XRCC1 399Gln may indicate favorable overall survival (GlnGln + GlnArg vs ArgArg: HR = 0.65(95%CI: 0.43–0.98)) and favorable PFS (GlnGln vs ArgArg: HR = 0.72(95%CI: 0.48–0.97)) in Asian patients; while in Caucasian patients, XRCC1 399Gln indicated poorer overall survival (GlnGln vs ArgArg: HR = 2.29(95%CI: 1.25–3.33)).

**Conclusions:**

Our results indicated that in NSCLC patients treated with platinum-based regimen, XRCC1 194Arg allele suggest poor objective response rate, the GlnGln genotype of XRCC1 399 suggest poorer overall survival in Caucasian patients, and longer PFS in Asian patients.

**Electronic supplementary material:**

The online version of this article (doi:10.1186/s12885-017-3487-y) contains supplementary material, which is available to authorized users.

## Background

Lung cancer, with growing incidence, is becoming one of the most prevalent cancer types all over the world. And it’s the leading cause of cancer death in males and second leading cause of cancer death in females, approximate 17.6% of the cancer deaths was due to lung cancer. [1] It usually develop silently, with non-specific clinical symptoms in the early period, and is apt to be neglected, most patients developed to advanced stage when they had some symptoms, and lost the opportunity of radical surgery. [2] For decades, platinum-based combination chemotherapy has been established as the cornerstone of advanced non-small cell lung cancer (NSCLC) treatment [3, 4]. Although molecular-targeted therapy has been confirmed as first-line therapy option for those advanced NSCLC with driver gene mutations, including epidermal growth factor receptor (EGFR), anaplastic lymphoma receptor tyrosine kinase (ALK), and KRAS mutations in recent years, still majority of NSCLC patients are not indicated to adopt molecular-targeted therapy. For these patients, platinum-based combination remains the first choice. But some NSCLC patients benefit from the treatment, while others failed. That means, not all the advanced NSCLC patients can benefit from platinum-based chemotherapy. In the new era, it is very important to select suitable treatment program for individualized treatment.

Anti-tumor mechanism of cisplatin and carboplatin is generally acknowledged as follows: cisplatin and carboplatin enter cell nucleus, bind to DNA and form DNA adducts which lead to intrastrand or interstrand cross-links, result in DNA synthesis/replication dysfunction and DNA structure disruption, which ultimately brings about cell proliferation inhibition and cell apoptosis. [5, 6] Resistance to platinum agents is suggested to be the main reason for treatment failure. One proposed mechanism of platinum resistance is attributed to enhanced function of DNA repair system, which can repair and rescue the damaged DNA and help tumor cells survive. [7, 8] In other words, DNA repair pathway plays an important role in the treatment response to the platinum-based chemotherapy of NSCLC patients.

Base excision repair (BER) pathway is a DNA repair pathway that repairs damaged DNA throughout the cell cycle, and it is important in carcinogenesis and in response to DNA-damaging chemotherapy. X-ray repair cross-complementing protein 1 (XRCC1), which located on chromosome no. 19q13.2–13.3, undertook the DNA repair mission of single-strand breaks formed by ionizing radiation and alkylating agents. This protein interacts with DNA ligase III, polymerase beta and poly (ADP-ribose) polymerase, and forms a repair complex to participate in the BER pathway [9–12].

So far, a number of studies have investigated the role of XRCC1 polymorphisms with clinical outcomes of advanced NSCLC treated with platinum-based chemotherapy, but the results were quite controversial, some studies supported that there were some association between XRCC1 polymorphisms and clinical outcomes of advanced NSCLC treated with platinum-based chemotherapy (treatment response(TR), overall survival(OS) or progression-free survival(PFS)), [13–28] but others had different views. [29–31] To explore the association between XRCC1 polymorphisms with clinical outcomes of advanced NSCLC treated with platinum-based chemotherapy, we performed this meta-analysis under the Preferred Reporting Items for Systematic Reviews and Meta-Analyses (PRISMA) statement guidelines [32].

## Methods

We carried out this meta-analysis based on the PRISMA statement, all data was extracted from published papers, so that ethical approval was not required per our institutional ethics committee.

## Search strategy and selection criteria

Eligible studies were identified by searching the PubMed, CNKI, EBSCO and Cochrane databases (prior to July 2015) using “XRCC1” or “X-ray repair cross-complementing protein 1”, “lung”, “polymorphism” and “platinum”. Additional articles were identified through the reference cited in the first series of articles selected. Only research articles with human subjects were included and the language was not limited. The included studies have to be designed to evaluate the XRCC1 polymorphisms and clinical outcomes of advanced NSCLC (no opportunity of surgery) treated with platinum-based chemotherapy. And a study was excluded if any of the following cases occurred: (i) the study did not report any clinical outcome; (ii) studies using XRCC1 polymorphisms either to predict lung cancer’s risk or to predict treatment toxicity; (iii) studies reported with the same data or overlapping data by the same authors; (iv) the response rate or overall survival reported in the study was either not specific to polymorphism or could not be attributed to a specific polymorphism; (v) the response rate or overall survival stratified by SNP was neither reported in nor derivable from the original article, and the principal investigator declined or was unable to provide this information on request.

### Data extraction

Upon carefully reading through all articles of eligible studies, information was carefully extracted. For each study, characteristics such as name of first author, year of publication, original country and ethnicity of the patients, tumor stage, sample size (case no.), genotyping method, genotype distribution and clinical outcomes were collected. For studies including different subpopulations according to ethnicity or experiment design, we considered each subpopulation as a separate study in the meta-analysis. For example, the study carried out by Liao et al. [20] included training set and validation set two subpopulations, each set was used as a separate study in the meta-analysis. Ethnicity was categorized as “Caucasian” (European descendants) and “Asian”(mainland China, Taiwan and Korea).

### Statistical analysis

The strength of association between XRCC1 polymorphisms and response rate of advanced NSCLC treated with platinum-based chemotherapy was assessed by Crude odds ratios (ORs) with the corresponding 95% confident intervals (CIs). [33] The odds of response rate were defined as the ratio of complete or partial response against stable or progressive disease [CR + PR vs. PD + SD, evaluated by the WHO criteria or the Response Evaluation Criteria in Solid Tumors criteria (RECIST)]. The pooled ORs were performed for an allele comparison (C vs. Y), a homozygous model (CC vs. YY), a heterozygous model (CY vs. YY), a recessive model (CC vs. CY + YY) and a dominant model (CC + CY vs. YY). [33] Overall survival (OS) and progression free survival (PFS) were evaluated by calculating pooled Cox proportional hazard ratios (HRs) and 95% confidence intervals (CIs) as relevant effect measures, HRs and 95% CIs were obtained directly from the raw data, or indirectly from the Kaplan-Meier curve of an article. [34] Q test and *I* ^*2*^ test were used for the assessment of heterogeneity. The fixed effects model was applied when the effects were assumed to be homogenous (Q test shown *P* value >0.1), otherwise a random effects model was applied for meta-analysis. When heterogeneity was present, and the number of the studies included was large enough to perform the multivariable regression analysis, a meta-regression analysis was employed to explore the sources of heterogeneity. Furthermore, subgroup analyses were conducted by ethnicity and sample size (*n* > 100). Hardy-Weinberg Equilibrium (HWE) were assessed by http://www.oege.org/software/hwe-mr-calc.shtml [35] on September 15, 2015. Begg’s funnel plots and Egger’s linear regression test were used to estimate publication bias. Sensitivity analysis was performed to evaluate the stability of the results in the procedure of re-analysis after interchange of fixed or random effects model and omitting each study one at a time, especially small sample studies.

All the statistical analyses were performed using STATA version 12.0 (STATA Corporation, College Station, TX).

## Results

**Fig. 1:**
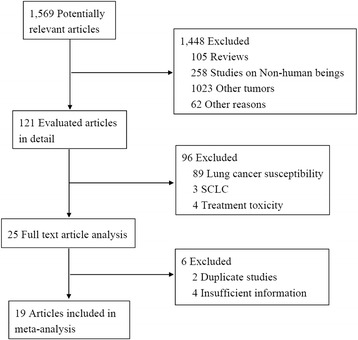
Literature search and selection of included studies

Overall 1569 potential studies were selected during the first step of systematic literature review, and a further review of the searched trials excluded 1448 studies, including 105 review articles, 258 studies on non-human beings, 1023 studies on other tumors, and 62 studies for other reasons. The remaining 121 studies were evaluated further, 96 studies were excluded, including 89 studies on lung cancer susceptibility, 3 studies on SCLC, 4 studies on treatment toxicity. Through detailed assessment, in the end, 19 follow-up studies were considered to meet all in inclusion criteria (Fig. 1). These were included in final analyses.

### Study characteristics

A total of 19 studies including 2815 individuals were eligible for the final analysis, in which 18 studies (2815 individuals) were eligible for XRCC1 399 analysis, and 8 studies (1208 individuals) were eligible for XRCC1 194 analysis. Table 1 listed the main characteristics and genotype distribution of XRCC1 399 (rs 25487) and XRCC1 194 (rs 1799782) with respect to response rate and overall survival rate, including first author, published year, ethnicity, original country, source of controls and genotype distribution. Five of these studies were conducted on Caucasian patients, and 14 were conducted on Asian patients. Fifteen were published in English-language journals. Four were published in Chinese-language journals. The sample size of each report ranged from 45–382 individuals.

**Table 1:** Main characteristics of studies included in this meta-analysis

Study	Year	Country	Ethnicity	Case No.	Genotyping method	Stage	Treatment Response (Genotype distribution)												Outcome	SNPs of XRCC1	HWE
							XRCC1 Arg399Gln						XRCC1 Arg194Trp								
							CR + PR			SD + PD			CR + PR			SD + PD					
							GG	GA	AA	GG	GA	AA	CC	CT	TT	CC	CT	TT			
Peng [17]	2014	China	Asian	235	PCR-CTTP	IIIA-IV	40	41	3	71	74	6	−	−	−	−	−	−	TR/OS/PFS	Arg399Gln	<0.005
Zhang [23]	2014	China	Asian	375	Sequenom MassARRAY	IIIA-IV	49	54	24	125	94	29	60	44	23	118	90	41	TR/OS/PFS	Arg194Trp Arg399Gln	<0.05
Lee [24]	2013	S. Korea	Asian	382	Sequenome mass spectrometry-based genotyping assay	III-IV	110	64	5	100	75	16	−	−	−	−	−	−	TR/OS	Arg399Gln	>0.05
Tiseo [30]	2013	Italy	Caucasian	93	TaqMan	IIIB-IV	14		4	27		2	−	−	−	−	−	−	TR/OS	Arg399Gln	−
Zhao [26]	2013	China	Asian	147	TaqMan allelic discrimination assay	IIIA-IV	21	24	8	56	31	5	32	20	1	51	35	6	TR/OS/PFS	Arg194Trp Arg399Gln	>0.05
Li [18]	2012	China	Asian	89	PCR-RFLP	IIIA-IV	20	6		24	39		−	−	−	−	−	−	TR	Arg399Gln	−
Liao 1[20]	2012	Taiwan	Asian	62	SNPstream UHT	IIIB-IV	9	9	1	17	22	4	−	−	−	−	−	−	TR/OS	Arg399Gln	>0.05
Liao 2[20]	2012	Taiwan	Asian	45	SNPstream UHT	IIIB-IV	−	−	−	−	−	−	−	−	−	−	−	−	OS	Arg399Gln	>0.05
Joerger [22]	2012	Netherlands	Caucasian	131	BigDye Terminator Cycle Sequencing Ready Reaction Kit	IIIB-IV	17	18	5	34	45	12	−	−	−	−	−	−	TR/OS/PFS	Arg399Gln	>0.05
Xu [14]	2011	China	Asian	130	PCR-RFLP	IIIB-IV	30	14	0	36	40	10	12	14	18	42	26	18	TR	Arg194Trp Arg399Gln	>0.05
Zhou [21]	2011	China	Asian	111	PCR-RFLP	IV	29	6		42	34		−	−	−	−	−	−	TR/TTP	Arg399Gln	−
Yuan [31]	2010	China	Asian	199	PCR-RFLP	IIIA-IV	**−**	**−**	**−**	**−**	**−**	**−**	−	−	−	−	−	−	OS/PFS	Arg194Trp Arg399Gln	−
Hong [13]	2009	China	Asian	164	PCR-RFLP	III-IV	26	28	3	44	53	10	19	31	7	54	42	11	TR	Arg194Trp Arg399Gln	>0.05
Kalikaki [19]	2009	Greece	Caucasian	119	PCR-RFLP	IIIA-IV	11	26		21	60		−	−	−	−	−	−	TR/OS	Arg399Gln	−
Sun [27]	2009	China	Asian	87	3D DNA microarray	IV	14	8	1	39	22	3	8	18	5	31	19	6	TR	Arg194Trp Arg399Gln	>0.05
Yao [29]	2009	China	Asian	102	PCR-RFLP	IIIB-IV	13		9	32		48	−	−	−	−	−	−	TR/OS	Arg399Gln	−
De las Penas R. [25]	2006	Spain	Caucasian	135	allelic discrimination assay	IIIB-IV	-	−	−	−	−	−	−	−	−	−	−	−	OS	Arg399Gln	−
Yuan [16]	2006	China	Asian	200	PCR-RFLP	Advanced							24	38	10	69	46	13	TR	Arg194Trp	>0.05
Gurubhagavatula [28]	2004	USA	Caucasian	103	PCR-RFLP	IIIA-IV	-	−	−	−	−	−	−	−	−	−	−	−	OS	Arg399Gln	-
Wang [15]	2004	China	Asian	105	PCR-RFLP	IIIB-IV	22	9	2	31	33	8	11	19	3	43	18	11	TR	Arg194Trp Arg399Gln	>0.05

**Footnote:** *TR* treatment response, *OS* overall survival, *PFS* progression free survival, *CR* complete response, *PR* partial response, *SD* stable disease, *PD* progressive disease, *SNPs* single nucleotide polymorphisms, *XRCC1* X-ray repair cross-complementing protein 1, *HWE* Hardy-Weinberg equilibrium, G = Arg, A = Gln, C = Arg, T = Trp

### Meta-analysis results

### XRCC1 194 polymorphism

#### Objective response

**Fig. 2:**
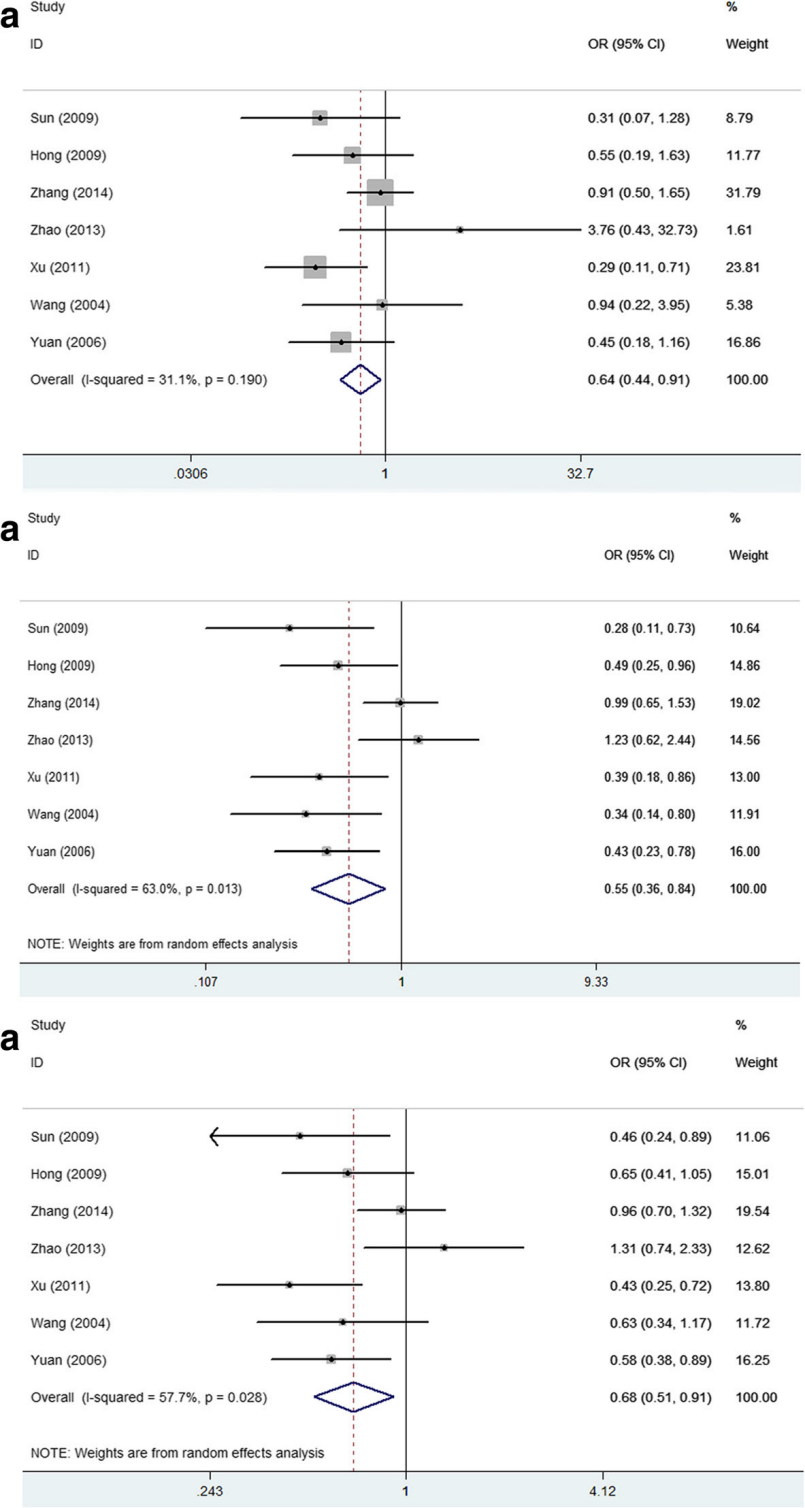
Forest plots of XRCC1 Arg194Trp polymorphisms and objective response in platinum-based chemotherapy by different allele contrast models. **a** homozygous model, **b** recessive model, **c** allele comparison. An OR >1 (or <1) indicates that the 194Arg is more (or less) likely to show response than 194Trp

Seven studies including 1208 individuals referred the predictive value of XRCC1 Arg194Trp with respect to the sensitivity of lung cancer to platinum-based treatment. All the studies were carried out based on Asian population. In the homozygous model, the Arg genotype was inverse associated with objective response in all patients (ArgArg vs TrpTrp: OR = 0.64(95%CI: 0.44-0.91), *p* = 0.190, P_Begg_ = 0.368, P_Egger_ = 0.943, Fig. 2a). The ORs in homozygous model, dominant model and heterozygous model showed a gene dosage effect that Arg genotype is associated with worse response rates of platinum-based treatment (ArgArg vs TrpTrp: OR = 0.64(95%CI: 0.44-0.91), *p* = 0.190, P_Begg_ = 0.368, P_Egger_ = 0.943; ArgArg + TrpArg vs TrpTrp: OR = 0.79(95%CI: 0.56-1.11), *p* = 0.324, P_Begg_ = 0.230, P_Egger_ = 0.337; TrpArg vs TrpTrp: OR = 1.05(95%CI: 0.73-1.51), *p* = 0.347, P_Begg_ = 0.035, P_Egger_ = 0.088; Table 2). Recessive model also showed that the ArgArg genotype of XRCC1 194 was associated with worse objective response in all patients treated with platinum-basedregimen (ArgArg vs TrpArg + TrpTrp: OR = 0.55(95%CI: 0.36-0.84), *p* = 0.013, P_Begg_ = 0.072, P_Egger_ = 0.065; Fig. 2b). Analysis of allele comparison consistent with the results (Arg vs Trp: OR = 0.68(95%CI: 0.51-0.91), *p* = 0.028, P_Begg_ = 0.368, P_Egger_ = 0.317; Fig. 2c). This indicates that the 194Arg allele may be indicative of poorer response rates to platinum-based treatment than the 194Trp allele.

**Table 2:** The association between XRCC1 Arg194Trp and Arg399Gln polymorphisms and objective response, OS and PFS

XRCC1	Objective response			Overall survival			Progression-free survival		
	Studies(refs.)	Pooled OR	P_h_ /*I* ^2^(%)	Studies(refs.)	Pooled HR	P_h_ /*I* ^2^(%)	Studies(refs.)	Pooled HR	P_h_ /*I* ^2^(%)
**Arg194Trp**									
ArgArg vs. TrpTrp									
All	7 [13–16, 23, 26, 27]	***0.64(0.44-0.91)***	0.19/31.1	3 [23, 26, 31]	1.19(0.73–1.65)	0.92/0.0	3 [23, 26, 31]	1.28(0.74–1.82)	0.92/0.0
Sample size>100	6 [13–16, 23, 26]	***0.67(0.46-0.97)***	0.17/35.4	3 [23, 26, 31]	1.19(0.73–1.65)	0.92/0.0	3 [23, 26, 31]	1.28(0.74–1.82)	0.92/0.0
Year ≥ 2009	5 [13, 14, 23, 26, 27]	***0.65(0.44-0.99)***	0.09/49.8	3 [23, 26, 31]	1.19(0.73–1.65)	0.92/0.0	3 [23, 26, 31]	1.28(0.74–1.82)	0.92/0.0
TrpArg vs. TrpTrp									
All	7 [13–16, 23, 26, 27]	1.05(0.73–1.51)	0.35/10.8	-	-	-	-	-	-
Sample size>100	6 [13–16, 23, 26]	1.04(0.71–1.52)	0.24/25.4	-	-	-	-	-	-
Year ≥ 2009	5 [13, 14, 23, 26, 27]	0.91(0.60–1.39)	0.56/0.0	-	-	-	-	-	-
ArgArg + TrpArg vs. TrpTrp									
All	7 [13–16, 23, 26, 27]	0.79(0.56–1.11)	0.32/13.9	1 [26]	-	-	1 [26]	-	-
Sample size>100	6 [13–16, 23, 26]	0.80(0.57–1.14)	0.23/27.1	1 ([26])	-	-	1 [26]	-	-
Year ≥ 2009	5 [13, 14, 23, 26, 27]	0.75(0.51–1.10)	0.26/24.6	1 ([26])	-	-	1 [26]	-	-
ArgArg vs. TrpArg + TrpTrp									
All	7 [13–16, 23, 26, 27]	***0.55(0.36-0.84)***	0.01/63.0	3 [23, 26, 31]	1.07(0.79–1.35)	0.79/0.0	3 [23, 26, 31]	0.94(0.72–1.17)	0.648/0.0
Sample size>100	6 [13–16, 23, 26]	***0.60(0.39-0.92)***	0.02/62.7	3 [23, 26, 31]	1.07(0.79–1.35)	0.79/0.0	3 [23, 26, 31]	0.94(0.72–1.17)	0.648/0.0
Year ≥ 2009	5 [13, 14, 23, 26, 27]	0.63(0.37–1.05)	0.02/65.9	3 [23, 26, 31]	1.07(0.79–1.35)	0.79/0.0	3 [23, 26, 31]	0.94(0.72–1.17)	0.648/0.0
**Arg399Gln**									
GlnGln vs. ArgArg									
All	10 [13–15, 17, 20, 22–24, 26, 27]	0.77(0.38–1.57)	0.005/62.0	7 [17, 19, 22–24, 28, 31]	1.15(0.61–1.69)	0.006/66.6	5 [17, 22, 23, 26, 31]	***0.72(0.48-0.97)***	0.136/42.9
Sample size>100	8 [13–15, 17, 22–24, 26]	0.77(0.35–1.73)	0.002/69.9	7 [17, 19, 22–24, 28, 31]	1.15(0.61–1.69)	0.006/66.6	5 [17, 22, 23, 26, 31]	***0.72(0.48-0.97)***	0.136/42.9
Asian	9 [13–15, 17, 20, 23, 24, 26, 27]	0.75(0.33–1.68)	0.003/66.1	4 [17, 23, 24, 31]	0.86(0.41–1.30)	0.052/61.2	4 [17, 23, 26, 31]	***0.67(0.40-0.94)***	0.106/51.0
Caucasian	1 [22]	-	-	3 [19, 22, 28]	***2.29(1.25-3.33)***	0.423/0.0	1 [22]	-	-
Year ≥ 2009	9 [13, 14, 17, 22–24, 26, 27]	0.83(0.39–1.77)	0.005/63.5	6 [17, 19, 22–24, 31]	1.06(0.54–1.57)	0.011/66.2	5 [17, 22, 23, 26, 31]	***0.72(0.48-0.97)***	0.136/42.9
GlnArg vs. ArgArg									
All	10 [13–15, 17, 20, 22–24, 26, 27]	0.90(0.67–1.21)	0.053/46.2	7 [17, 19, 23–25, 28, 31]	0.93(0.68–1.18)	0.003/69.3	4 [17, 23, 26, 31]	0.96(0.79–1.14)	0.416/0.0
Sample size>100	8 [13–15, 17, 22–24, 26]	0.89(0.63–1.26)	0.02/57.8	7 [17, 19, 23–25, 28, 31]	0.93(0.68–1.18)	0.003/69.3	4 [17, 23, 26, 31]	0.96(0.79–1.14)	0.416/0.0
Asian	9 [13–15, 17, 20, 23, 24, 26, 27]	0.90(0.65–1.25)	0.035/51.8	4 [17, 23, 24, 31]	0.91(0.56–1.27)	0.003/78.1	4 [17, 23, 26, 31]	0.96(0.79–1.14)	0.416/0.0
Caucasian	1 [22]	-	-	3 [19, 25, 28]	1.01(0.51–1.51)	0.057/65.2	-	-	-
Year ≥ 2009	9 [13, 14, 17, 22–24, 26, 27]	0.97(0.79–1.20)	0.112/38.4	5 [17, 19, 23, 24, 31]	0.97(0.64–1.31)	0.003/74.7	4 [17, 23, 26, 31]	0.96(0.79–1.14)	0.416/0.0
GlnGln + GlnArg vs. ArgArg									
All	13 [13–15, 17–24, 26, 27]	0.72(0.50–1.04)	0.000/70.9	6 [17, 19, 20, 23, 26, 31]	0.73(0.50–1.05)	0.001/72.5	4 [17, 23, 26, 31]	0.94(0.77–1.10)	0.274/22.8
Sample size>100	10 [13–15, 17, 19, 21–24, 26]	0.78(0.53–1.16)	0.000/72.5	5 [17, 19, 23, 26, 31]	0.85(0.55–1.15)	0.02/65.9	4 [17, 23, 26, 31]	0.94(0.77–1.10)	0.274/22.8
Asian	11 [13–15, 17, 18, 20, 21, 23, 24, 26, 27]	0.70(0.46–1.07)	0.000/75.7	5 [17, 20, 23, 26, 31]	***0.65(0.43-0.98)***	0.003/71.7	4 [17, 23, 26, 31]	0.94(0.77–1.10)	0.274/22.8
Caucasian	2 [19, 22]	0.82(0.46–1.44)	0.966/0.0	1 [19]	-	-	-	-	-
Year ≥ 2009	12 [13, 14, 17–24, 26, 27]	0.93(0.65–1.34)	0.004/64.3	6 [17, 19, 20, 23, 26, 31]	0.73(0.50–1.05)	0.001/72.5	4 [17, 23, 26, 31]	0.94(0.77–1.10)	0.274/22.8
GlnGln vs. GlnArg + ArgArg									
All	12 [13–15, 17, 20, 22–24, 26, 27, 29, 30]	0.85(0.50–1.45)	0.028/49.0	5 [19, 24, 26, 29, 30]	1.19(0.53–1.84)	0.01/68.9	2 [26, 30]	0.94(0.37–1.52)	0.435/0.0
Sample size>100	9 [13–15, 17, 22–24, 26, 29]	0.78(0.43–1.41)	0.015/58.0	4 [19, 24, 26, 29]	***1.44(1.01-1.88)***	0.686/0.0	1 [26]	-	-
Asian	10 [13–15, 17, 20, 23, 24, 26, 27, 29]	0.75(0.41–1.37)	0.023/53.4	3 [24, 26, 29]	1.40(0.96–1.84)	0.999/0.0	1 [26]	-	-
Caucasian	2 [22, 30]	1.58(0.42–5.98)	0.194/40.6	2 [19, 30]	1.43(−1.05–3.91)	0.058/72.1	1 [30]	-	-
Year ≥ 2009	11 [13, 14, 17, 20, 22–24, 26, 27, 29, 30]	0.87(0.47–1.62)	0.038/51.0	5 [19, 24, 26, 29, 30]	1.19(0.53–1.84)	0.01/68.9	2 [26, 30]	0.94(0.37–1.52)	0.435/0.0

**Footnote:** *OR* odds ratio, *HR* hazard ratio, *refs* references, *OR/HR* with the corresponding 95% *CIs* >/<1 means significance

#### Overall survival and progression-free survival

Data from 3 included studies (including 721 patients) were applicable for the analysis. As shown in Table 2, there is no association between the 194Arg genotype and a significant increase of hazard for death in all patients (ArgArg vs TrpTrp: HR = 1.19(95%CI: 0.82–1.73), *p* = 0.90). The pooled results showed no significant association between XRCC1 Arg194Trp polymorphism and PFS under either kinds of genetic model (Table 2). No significant heterogeneity was detected among the predictive values.

### XRCC1 399 polymorphism

#### Objective response

**Fig. 3:**
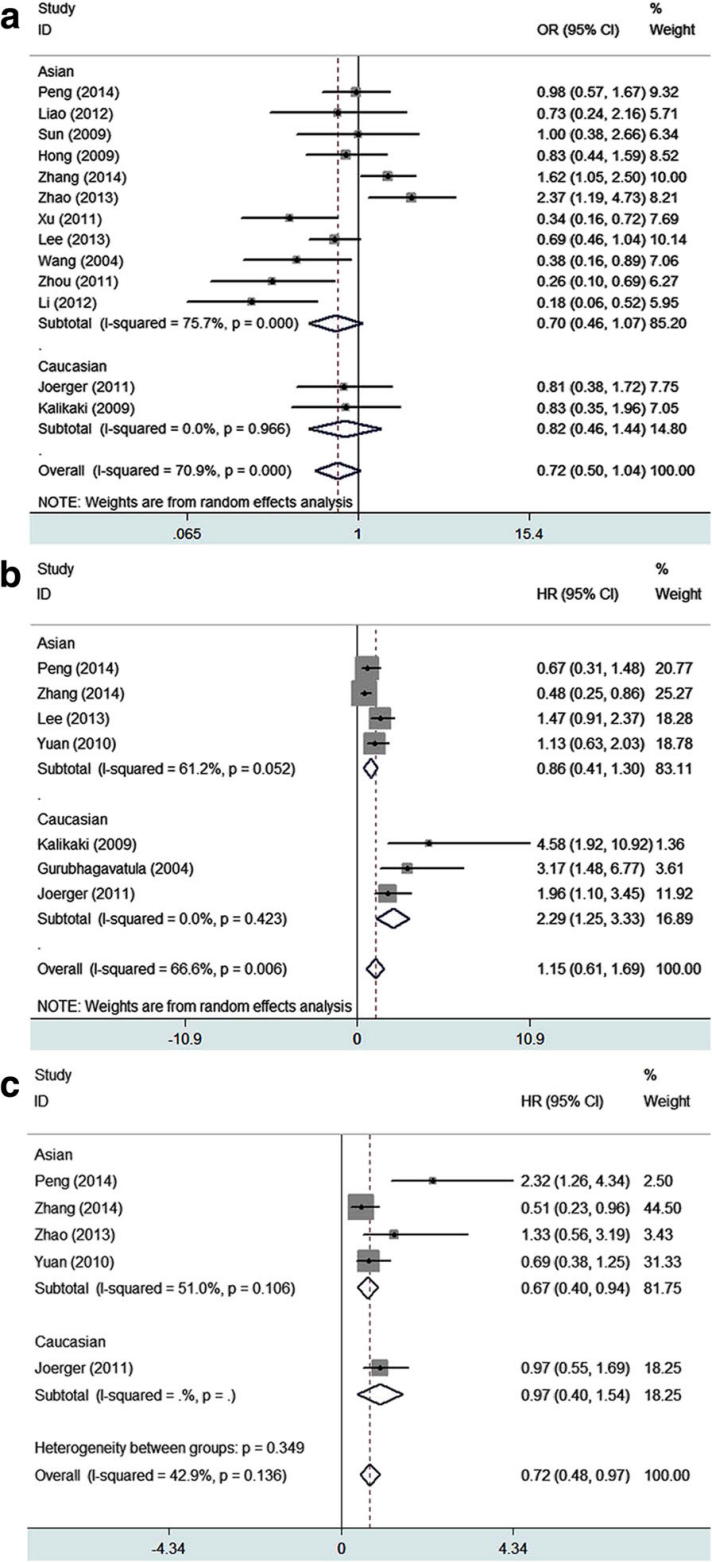
Forest plots of XRCC1 Arg399Gln polymorphisms and clinical outcomes in platinum-based chemotherapy. **a** Dominant model of association between 399Gln and objective response relative to 399Arg; An OR >1 (or <1) indicates that the 399Gln is more (or less) likely to show response than 399Arg. **b** Homozygous model of association between 399Gln and overall survival relative to 399Arg; An HR > 1 (or <1) indicates that the 399Gln is more (or less) likely to show worse overall survival than 399Arg. **c** Homozygous model of association between 399Gln and progression free survival relative to 399Arg. An HR > 1 (or <1) indicates that the 399Gln is more (or less) likely to show worse progression free survival than 399Arg

Eighteen studies including 2814 individuals were qualified for analyzing the association between XRCC1 Arg399Gln polymorphism and treatment response to platinum-based treatment of NSCLC. No significant association was found between the 399Gln allele and response rate relative to the 399Arg allele in either kinds of genetic model (Table 2). However, the results show a suspected borderline association between the 399Gln allele and poorer treatment response in dominant model (GlnGln + GlnArg vs ArgArg: OR = 0.72(95%CI: 0.50–1.04), *p* = 0.000, P_Begg_ = 0.077, P_Egger_ = 0.093; Fig. 3a). And stratified analysis by ethnicity showed that the borderline association mainly derived from Asian population. Sensitive analysis further confirms the robustness of our results, so we considered that there was no significant association between XRCC1 399Gln allele and the objective response rate relative to 399Arg allele.

#### Overall survival

**Fig. 4:**
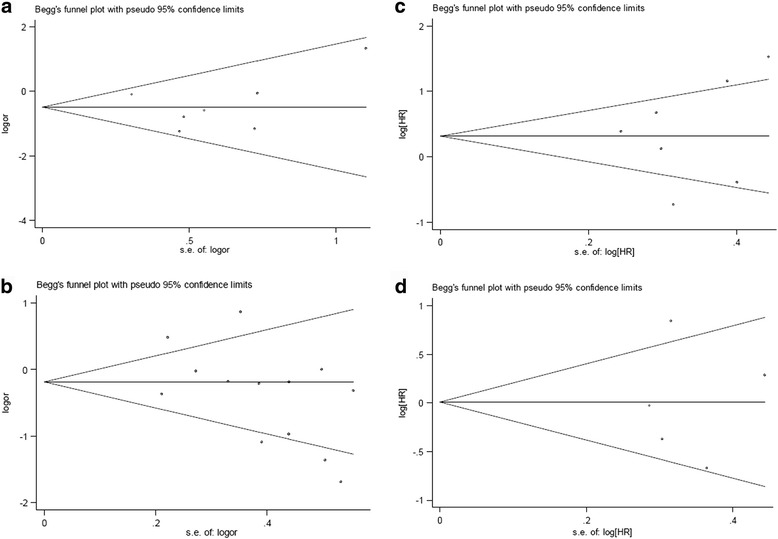
Begg’s funnel plot for publication bias test.Homozygous model of association between XRCC1 Arg194Trp and objective response (ArgArg vs TrpTrp); **a** Homozygous model of association between XRCC1 Arg399Gln and objective response (GlnGln vs ArgArg); **b** Homozygous model of association between XRCC1 Arg399Gln and overall survival (GlnGln vs ArgArg); **c** Homozygous model of association between XRCC1 Arg399Gln and PFS (GlnGln vs ArgArg)

Thirteen studies covering a total of 2128 patients were eligible for the analysis. The results show neither in dominant model nor in homozygous model, no association was detected between the 399Gln allele and overall survival. In dominant model (GlnGln + GlnArg vs ArgArg: HR = 0.73(95%CI: 0.50–1.05), *p* = 0.001, P_Begg_ = 0.133, P_Egger_ = 0.169; see Figure, Additional file 1); in homozygous model (GlnGln vs ArgArg: HR = 1.15(95%CI: 0.61–1.69), *p* = 0.006, P_Begg_ = 0.764, P_Egger_ = 0.594; Fig. 3b). Results of stratified analysis by ethnicity showed as follows: in dominant model, the GlnGln + GlnArg genotypes of XRCC1 399 indicated better overall survival than the ArgArg genotype in Asian patients treated with platinum-based regimen (GlnGln + GlnArg vs ArgArg: HR = 0.65(95%CI: 0.43–0.98), *p* = 0.003, P_Begg_ = 0.260, P_Egger_ = 0.178; see Figure, Additional file 2); and in homozygous model, the GlnGln genotype of XRCC1 399 showed no association with overall survival in Asian patients (GlnGln vs ArgArg: HR = 0.86(95%CI: 0.41–1.30), *p* = 0.052, P_Begg_ = 0.308, P_Egger_ = 0.287; Fig. 3b); while in Caucasian patients, the result showed the GlnGln genotype of XRCC1 399 was associated with poorer overall survival (GlnGln vs ArgArg: HR = 2.29(95%CI: 1.25–3.33), *p* = 0.423, P_Begg_ = 0.296, P_Egger_ = 0.045; Fig. 3b). No publication bias was detected according to the results of funnel plot and the Egger’s test (Fig. 4).

#### Progression-free survival

Only six studies (including 1180 individuals) were applicable for the analysis. The results show GlnGln genotype of XRCC1 399 was associated with longer PFS than ArgArg genotype in patients treated with platinum-based regimen (GlnGln vs ArgArg: HR = 0.72(95%CI: 0.48–0.97), *p* = 0.136; Fig. 3c). No publication bias was detected according to the results of funnel plot and the Egger’s test (GlnGln vs ArgArg: P_Begg_ = 1, P_Egger_ = 0.989; Fig. 4).

#### Sensitivity analysis

Examining genotype frequencies in the controls, significant deviation from HWE was detected in the two articles. [17, 23] When these studies were excluded, the conclusion remain unchanged in the meta-analysis. When the study of small sample (sample size ≤ 100) was excluded, the conclusion remain unchanged in the meta-analysis (Table 2). Additionally, we performed a sensitivity analysis on time of published years. We excluded the papers published before 2009, and performed meta-analysis, the conclusion remain unchanged. Detailed results were showed in Table 2.

## Discussion

Platinum-based combination chemotherapy remains the first-line treatment regimen for advanced NSCLC. However, platinum (cisplatin or carboplatin) may cause severe toxic side effect, such as gastrointestinal reaction, neutropenia, anemia, renal toxicity and hepatic toxicity ect. Studies were carried out to explore whether non-platinum-based chemotherapy could achieve comparable efficacy as platinum-based chemotherapy. [36, 37] Meta-analysis’ results show gemcitabine plus docetaxel (GD) acquired similar survival with platinum-based regimens in first-line treatment of advanced NSCLC, platinum-based regimens had an advantage in time to progression (TTP) and overall response rate (ORR) with more grade 3–4 nausea/vomiting, anemia, neutropenia and febrile neutropenia compared with GD. [38] Besides, patients with platinum resistance may not benefit from platinum-based chemotherapy.

Finding predictive markers to guide personalized treatment is essential. The XRCC1 polymorphisms have been widely investigated in lung cancer, and it was reported that different genotype of XRCC1 could predict different lung cancer risk, also it was reported that different genotype of XRCC1 could predict different clinical outcomes (different response rate to platinum-based regimen, different overall survival and different progression-free survival). Meanwhile, others might have different opinions. To explore the relationship between XRCC1 polymorphism and clinical outcomes to platinum-based regimen, and further guide our clinical strategic decision, we conduct this analysis.

Our results showed that XRCC1 194Arg allele was negatively associated with the objective response rate relative to 194Trp, and interestingly, results of homozygous model, dominant model and heterozygous model suggested a gene dosage effect negative correlation between 194Arg allele and objective response rate. This further confirms the robustness of our results. But no association was found between 194Arg allele and either overall survival or PFS. That may due to too few eligible studies. Hence, the conclusions drawn in this meta-analysis about the association between 194Arg allele and both overall survival and PFS should be cautiously considered. More studies need to be carried out and applied for further analysis.

Analysis showed that XRCC1 399Gln allele played different roles in different ethnicity. Although under fixed models, association was found between XRCC1 399Gln allele and not only overall survival, but also objective response rate and progress free survival. As heterogeneity was detected in the analysis of overall survival and objective response rate, after random model was adopted, no significant association was found between XRCC1 399Gln allele and the objective response rate relative to 399Arg allele. XRCC1 399Gln allele indicated better overall survival in Asian patients in dominant model; while in Caucasian patients, the GlnGln genotype of XRCC1 399 was associated with poorer overall survival in homozygous model. Furthermore, in homozygous model, 399GlnGln genotype was associated with longer PFS than 399ArgArg genotype in Asian patients treated with platinum-based regimen. According to the analysis results, it seemed that XRCC1 339Gln allele had contradictory results on different ethnic groups. Maybe there were other reasons that may influence the results of OS and PFS. Because we know that response rate more directly reflects pharmacogenomics roles, while OS and PFS may be influenced by many other factors, such as supportive care, dietary habits, living habits, constitutional factors and so on. In addition to those factors, more studies with much larger sample size are required to be able to draw more definitive conclusions.

HWE states that allele and genotype frequencies in a population will remain constant from generation to generation in the absence of other evolutionary influences. It is not uncommon that quality of studies may vary in meta-analysis of genetic association studies in genetic epidemiology. As reports showed that XRCC1 was associated with lung cancer risk, we considered that violation of HWE was not necessarily be excluded in this analysis, and furthermore, no genotyping error was detected in those studies.

Heterogeneity was detected in parts of the analysis, through random effects model, some of them generated a significant OR/HR results, and all the results were confirmed by sensitive analysis. The existence of heterogeneity indicated variability, which may have been caused by different characteristics, such as ethnicity, region, sample size, gender, method of genotyping used among patient populations. Hence, stratified analyses of subpopulations are needed to reduce such variability, and much larger studies should be undertaken to ensure sufficient statistical power.

Many proteins involved in DNA damage repair system have a role in repairing the cross links by platinum. Nucleotide excision repair (NER) and base excision repair (BER) pathway are major DNA repair systems. Besides XRCC1, other DNA base excision repair genes including OGG1, [17] APE1, [17, 39–41] XRCC3, [42] PARP1, [43] were reported to be associated with clinical outcomes in NSCLC treated with platinum-based regimen. Genes involved in NER system such as ERCC1 [41, 43–45], ERCC2(XPD), [44, 46] BAG1, [46] XPA, [47] were also reported to be associated with clinical outcomes in NSCLC treated with platinum-based regimen. In addition, association between BRCA1, [41, 45] MDR1, [48] eIF3a, [49, 50] PKM2, [51] and clinical outcomes in NSCLC treated with platinum-based regimen were also investigated. Li P’s research demonstrated the combined effects of BAG1 and XPD polymorphisms on chemotherapy sensitivity and survival in patients with advanced NSCLC. [46] Huang ZL’s study showed the expression of ERCC1 and BRCA1 was significantly associated with the disease free survival (DFS) time in patients with NSCLC treated with adjuvant cisplatin-based chemotherapy, respectively. The combination of the ERCC1 and BRCA1 expression levels may be a promising prognostic prediction for adjuvant cisplatin-based chemotherapy. [45].

DNA repair genetic polymorphisms may be better used in the future to predict clinical outcomes from treatments in cancer care [52] and help to improve therapeutic regimen plan setting and patient care. More studies with much larger sample size are required to be able to draw definitive conclusions about the role of DNA repair variants and treatment outcome.

Although interesting results have been achieved in this meta-analysis, there are several limitations. First, a systematic review should ideally be conducted using individual patient data. However, it’s not practical because individual patient data from studies are not always easily obtainable, lacking of the original data of the included studies limited our further evaluation of potential interactions. Second, we only included the studies published in English and Chinese, studies published in other languages were difficult to get. Last, our results were based on unadjusted published estimates. Without data limitations, we could adjust them such as age, smoking condition, pathological type, gender et al., and get more definitive and detailed conclusions. Overall, our results indicated that in NSCLC patients treated with platinum-based regimen, XRCC1 194Arg allele suggest poor objective response rate, the GlnGln genotype of XRCC1 399 suggest poorer overall survival in Caucasian patients, and longer PFS in Asian patients.

## Additional files

Additional file 1: Dominant model of association between 399Gln and overall survival relative to 399Arg. (DOCX 73 kb)

Additional file 2: Dominant model of association between 399Gln and overall survival relative to 399Arg in Asian population. (DOCX 62 kb)

## Abbreviations

BER: Base excision repair
CI: Confidence interval
CR: Complete response
HR: Hazard ratio
HWE: Hardy-Weinberg Equilibrium
NSCLC: Non-small cell lung cancer
OR: Odds ratio
OS: Overall survival
PD: Progressive disease
PFS: Progression free survival
PR: Partial response,
PRISMA: Preferred Reporting Items for Systematic Reviews and Meta-Analyses
RCT: Randomized controlled trial
RECIST: Response Evaluation Criteria in Solid Tumors criteria,
RR: Risk ratio
SCLC: Small cell lung cancer
SD: Stable disease
SNP: Single nucleotide polymorphisms
TR: Treatment response
XRCC1: X-ray repair cross-complementing protein 1
