# Supplementary material for: Association between the XRCC1 polymorphisms and clinical outcomes of advanced NSCLC treated with platinum-based chemotherapy: a meta-analysis based on the PRISMA statement
Source: BMC Cancer. 2017 Jul 25;17:501. doi: 10.1186/s12885-017-3487-y (PMC5526237; doi:10.1186/s12885-017-3487-y)

**Additional file 1**

Dominant model of association between 399Gln and overall survival relative to 399Arg.


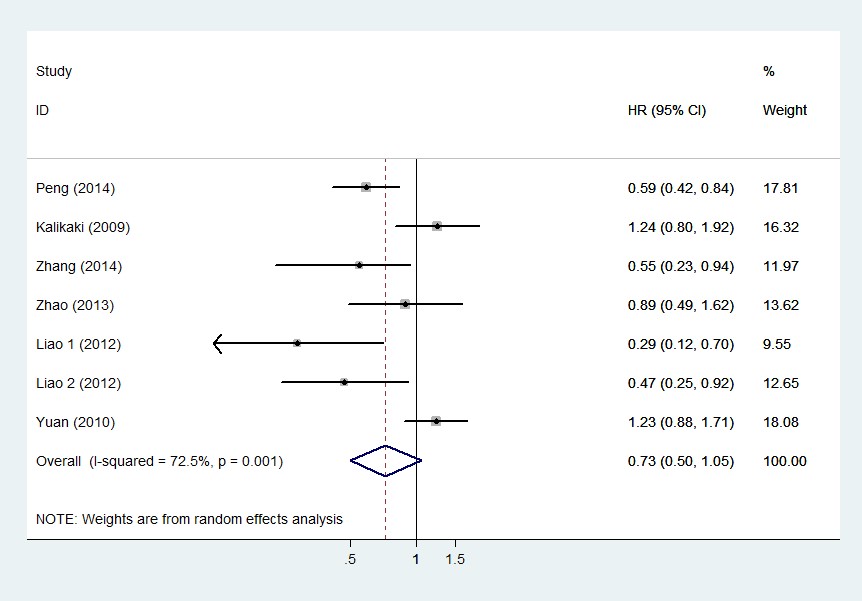

Supplement: Supplementary file 1 — Dominant model of association between 399Gln and overall survival relative to 399Arg. (DOCX 73 kb) [file 12885_2017_3487_MOESM1_ESM.docx]
